# Molecular identification and prevalence of trypanosomes in cattle distributed within the Jebba axis of the River Niger, Kwara state, Nigeria

**DOI:** 10.1186/s13071-021-05054-0

**Published:** 2021-10-29

**Authors:** Issa Funsho Habeeb, Gloria Dada Chechet, Jacob K. P. Kwaga

**Affiliations:** 1 Department of Biochemistry, Faculty of Life Sciences, Ahmadu Bello University Zaria, Zaria, Nigeria; 2 Department of Veterinary Public Health and Preventive Medicine, Faculty of Veterinary Medicine, Ahmadu Bello University, Zaria, Nigeria; 3 Africa Centre of Excellence for Neglected Tropical Diseases and Forensic Biotechnology, Ahmadu Bello University, Zaria, Nigeria; 4 National Space Research and Development Agency, Abuja Nigeria (NASRDA), Abuja, Nigeria

**Keywords:** Trypanosomiasis, Cattle, ITS-1, Prevalence, Jebba, Geospatial distribution, River Niger

## Abstract

**Background:**

Trypanosomiasis is a fatal disease that threatens the economy of at least 37 countries in sub-Saharan Africa, particularly with regard to livestock farming. In this study, we investigated the prevalence of trypanosome infection in cattle, and molecularly identified the species of trypanosomes in infected cattle and the spatial distribution of trypanosome-infected herds along the Jebba axis of the River Niger.

**Methods:**

A randomized cross-sectional study was conducted along the Jebba axis of the River Niger by screening cattle from 36 herd clusters by nested PCR using ITS-1 generic primers. Data generated were analysed using the Chi-square test at a 95% confidence interval.

**Results:**

Microscopic examination revealed three infected cattle out of 398 examined, representing 0.8% prevalence. Twelve animals (3.0%) were positive by PCR. Our results showed a decline in the packed cell volume of infected animals (24.7%). The infection rates were categorized as single infection in 11/12 (91.7%) and mixed infection in 1/12 (8.3%). Animals were most frequently infected by *Trypanosoma congolense* (50.0%), with *T. congolense* Savannah being the most prevalent subspecies (71.4%). Aside from the infection rate by age (10.0%) and relative distance of animals from the River Niger (56.2%), statistical differences in every other parameter tested were based on mere probabilistic chance. Spatial data showed that the disease was prevalent among herds located less than 3 km from the River Niger.

**Conclusions:**

Six species of trypanosomes were identified in cattle herds along the Jebba axis of the River Niger, with *T. congolense* being the most prevalent. Age and relative distance of herds from the River Niger may be risk factors for trypanosome infection in cattle herds in this area.

**Graphical abstract:**

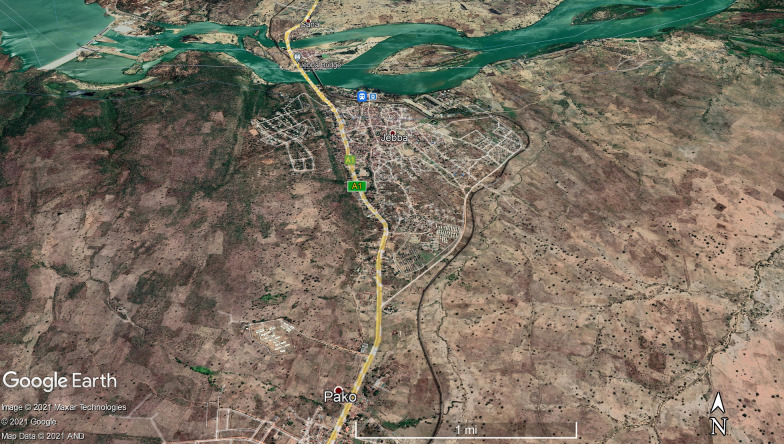

**Supplementary Information:**

The online version contains supplementary material available at 10.1186/s13071-021-05054-0.

## Background

African trypanosomiasis (AT) is a parasitic disease of public health concern affecting humans (human African trypanosomiasis) and animals (animal African trypanosomiasis), thereby limiting agricultural productivity in most developing countries in sub-Saharan Africa (sSA). The disease is caused by protozoan parasites belonging to the genus *Trypanosoma*. It is cyclically transmitted by tsetse flies and mechanically by other blood-feeding insects [1, 2]. Reports have shown that over 33% of Africa’s landmass has been pervaded by more than 30 *Glossina* species and subspecies [2, 3]. In Africa, the disease affects around 100 million head of cattle, with 6 million estimated in Nigeria alone out of a cattle population that is presently estimated at 20 million [4]. The World Health Organization (WHO) has estimated that 50–70 million individuals are at risk of tsetse bites in Africa, with about 30,000 reported cases per annum [3]. However, WHO-led intervention efforts targeting the elimination of HAT have resulted in a progressive decline in this incidence [5]. Nevertheless, no fewer than 1000 cases are being reported each year, despite continued active and passive screening efforts. The disease advances rapidly from a less virulent to a chronic form, and accounts for an economic loss of $4–5 billion in terms of gross domestic product [6, 7].

The major species responsible for animal African trypanosomiasis include *Trypanosoma congolense*, *T. brucei* and *T. vivax* [8]. In humans, *T. brucei gambiense* and *T. brucei rhodesiense* cause acute and chronic infections respectively [9]. However, several reports show the existence of atypical human infections caused by animal trypanosomes [10–14]. Therefore, identification of *Trypanosoma* species is fundamental to measuring the general threat posed by trypanosome species in animals and humans [15, 16].

There have been several recent developments in the diagnosis of AT towards understanding the disease transmission and distribution which is necessary for any successful treatment [17]. These advances have enabled accurate detection and identification of previously unidentified zoonotic *Trypanosoma* species [18]. While enzyme-based immunosorbent assay (ELISA) was considered an improvement with regard to the sensitivity of pathogen determination, antigen detection using monoclonal antibody (mAb)-based ELISA is quite unreliable due to the presence of immune active agents in the blood which cannot distinguish between active and previous infections [19].

The advent of polymerase chain reaction (PCR) as a novel technique for direct parasite detection and identification offered greater sensitivity and reliability than methods previously used. The use of *Trypanosoma* species-specific DNA probes [20, 21] and PCR analysis [22–25] has significantly enhanced the efficiency of identification and understanding of trypanosome diversity, especially the high prevalence of mixed trypanosome infections which exist in the field [26–28]. Consequently, with specific PCR tests using generic primers [15], the identification of 11 known tsetse-transmitted trypanosome species/subspecies as well as unknown species has been made possible through amplification of the ribosomal RNA gene loci of the internal transcribed spacer (ITS-1) region of the trypanosome genome due to its variable inter-species length and high copy number [29, 30].

Several studies on trypanosomiasis transmission have been conducted in some parts of Nigeria [10, 11, 22]. However, to date, there is no published report on the prevalence of AAT in Kwara state. In this study, we investigated the prevalence of trypanosome infection in cattle and molecularly identified the species of trypanosomes in infected cattle and the spatial distribution of trypanosome-infected herds along the Jebba axis of the River Niger.

## Methods

### Study area

This study was conducted along the Jebba axis of the River Niger, Kwara state. Jebba is located at the geographical coordinates 9°9′14″N, 4°48′43″E, with views of the River Niger. In light of its area, it is known as the “midland” and the “door” between the southern and northern parts of Nigeria [31]. According to the 2006 census, the city’s population in 2006 was 22,411, and it is approximately 500 km from Abuja, and 306 km and 600 km from Lagos and Kaduna, respectively.

### Study population

Our study population consisted of mostly transhumance cattle, having the possibility of mixing with sentinel animals. For parasite identification, animals with recent (≤ 2 weeks) administration of trypanocidal drugs were excluded from the study.

### Study design

A cross-sectional study was undertaken to capture cattle distribution across the Jebba axis of the River Niger, Kwara state, and its tributaries to assess trypanosome distribution across the geographical area in June 2019.

### Sampling method and sample size

A systematic random sampling techniques was employed in this study. Herds of cattle in each coordinate were pooled and considered as a cluster from which animals were sampled by systematic randomization. The sampling frame was identified by listing herd locations across Jebba, and samples were obtained in each cluster based on proportionto size of herd; i.e. 6% of cattle in each herd. A systematic random sampling technique was used to select animals in each of the randomized clusters, whereby the sampling interval was generated by dividing the herd size by the sample size required for that herd. The first study subject was randomly selected from among the cattle, while others were selected at an interval based on herd size [32]. Due to the complete absence of previous records on the prevalence of trypanosomiasis in our study area, 50% prevalence was assumed and the sample size was estimated [33]. A minimum total sample size of 384 cattle was drawn from across the identified clusters. Sample size calculation was based on a 95% confidence level, 50% assumed prevalence and 0.05 tolerable error. In each cluster, animals were considered in randomization regardless of health status so as to give an overall current infection status of the herd. Animals aged one (1) year and younger were considered young calves, while those over one (1) year were regarded as adults. Dentition was used to determine the ages of animals, and body condition scores (BCS) were assessed and adequately scored. Other parameters including breed, sex, source and location of the cattle were recorded. Herd data were collected to include the residency, travel history, herd size, history of trypanocidal treatment and disease history.

### Sample collection and parasitological analyses

Five millilitres of blood were collected from the jugular vein of each randomized animal using a sterile Vacutainer needle into tubes containing anticoagulant ethylenediaminetetraacetic acid (EDTA) [7]. Each sample was identified by a unique barcode system that corresponds to the name of the village, herd cluster and sample number. Samples were transported in an ice box to the laboratory and stored at 4 °C prior to laboratory analysis. Parasitological examination was done in the laboratory using the standard trypanosome detection methods, i.e., the buffy coat method (BCM) [32], haematocrit centrifugation technique (HCT) [34], parasite load estimation [35] and Giemsa-stained thick and thin films [36]. The packed cell volume (PCV) of each animal was also determined and the parasites were identified [37, 38].

### DNA extraction and PCR

The Quick-gDNA™ MiniPrep kit (Zymo Research Corporation, Irvine, CA, USA) was used for genomic DNA (gDNA) extraction from the blood as prescribed by the manufacturer. DNA yield and purity assessment were performed using a NanoDrop ND-100 UV spectrophotometer (NanoDrop Technologies, Inc./Thermo Fisher, Waltham, MA, USA) and the eluted DNA were stored at −20 °C until further use [32]. PCR amplification were carried out as described elsewhere [15], with slight modification. In the first round of the reaction, 3 µl of DNA was added to the PCR reaction mixture. PCR was performed in a total reaction volume of 25 µl containing 2.5 µl of standard Taq buffer (10×), 1.0 µl of dNTPs (10 mM), 1.0 µl of each primer (25 µM), 0.5 µl Taq DNA polymerase (5000 U/ml), 3.0 µl DNA template and 16.0 µl of nuclease-free water to make a final volume of 25 µl. The PCR runs were set as follows: initial denaturation at 95 °C for 2 min; 35 cycles of 30 s at 94 °C, 30 s at 54 °C and 60 s at 72 °C; final elongation for 5 min at 72 °C. In the first run of the nested PCR, Tryp 3 (forward, 5′TGCAATTATTGGTCGCGC3′) and 4 (reverse, 5′CTTTGCTGCGTTCTT3′) were used as outer primers, followed by Tryp 1 (forward, 5′AAGCCAAGTCATCCATCG3′) and 2 (reverse, 5′TAGAGGAAGCAAAAG3′), which served as inner primers in the second reaction run. From the first run, 2.0 µl of the PCR products was added to 23 µl of the mixture in the second round of the reaction in a fresh PCR tube. Cycling conditions were the same as the standard PCR except for a 1 °C increase in annealing temperature in the second reaction. A positive control was used in the validation experiment, and a negative control was included in each run [39]. The positive control was DNA extracted from a blood sample of an animal positive by microscopy, whereas the negative control was DNA from a non-infected blood sample. The amplified DNA was resolved on 2% agarose gel, visualized under a UV transilluminator and photographed with a gel documentation apparatus (Bio-Rad Molecular Imager Gel Doc System 170-8170 v.3) for clear visualization and reference purposes.

### DNA sequencing and phylogenetic analysis

Twenty microliters of samples positive by PCR were sequenced using the BigDye^®^ Terminator v3.1 Cycle Sequencing Kit (Applied Biosystems, Foster City, CA, USA), according to the manufacturer’s instructions. The sequences obtained were viewed on the FinchTV v.1.4.0 trace viewer (Applied Biosystems), while flanking regions of high signal-to-noise ratio were trimmed off the sequence to improve the accuracy and precision of sequence data obtained. Ambiguous nucleotides were edited and replaced with conventional ones based on the highest peaks recorded on the electropherogram. Each edited sequence was BLAST-searched against the DNA sequence database (NCBI) and/or the published databases for various trypanosome species (TriTrypDB), and isolates with GenBank hits of 80% or more were considered similar. Sequences of the ITS-1 region were aligned using ClustalW algorithm against known sequences in order to confirm species identity. MEGA (Molecular Evolutionary Genetics Analysis) version 7.0.2.6 was used to construct a phylogenetic tree using the maximum likelihood algorithm with 1000 bootstrap replicates to observe their evolutionary trend and variation over time.

### Statistical analysis

The results obtained from this study were subjected to descriptive statistics to determine the frequency and distribution of trypanosome infection across the study area. The prevalence among localities, breeds of cattle, and age and sex of the animals was expressed as percentages of the total number of animals sampled. This was done by dividing the number of infected animals by the total number of animals examined and expressed as percentages. Categorical values were evaluated using the Chi-square test to measure the strength between variables at a 95% confidence interval. All data obtained were analysed using SPSS statistical software version 20.0. Values of *P* < 0.05 were considered significant.

## Results

**Fig. 1:**
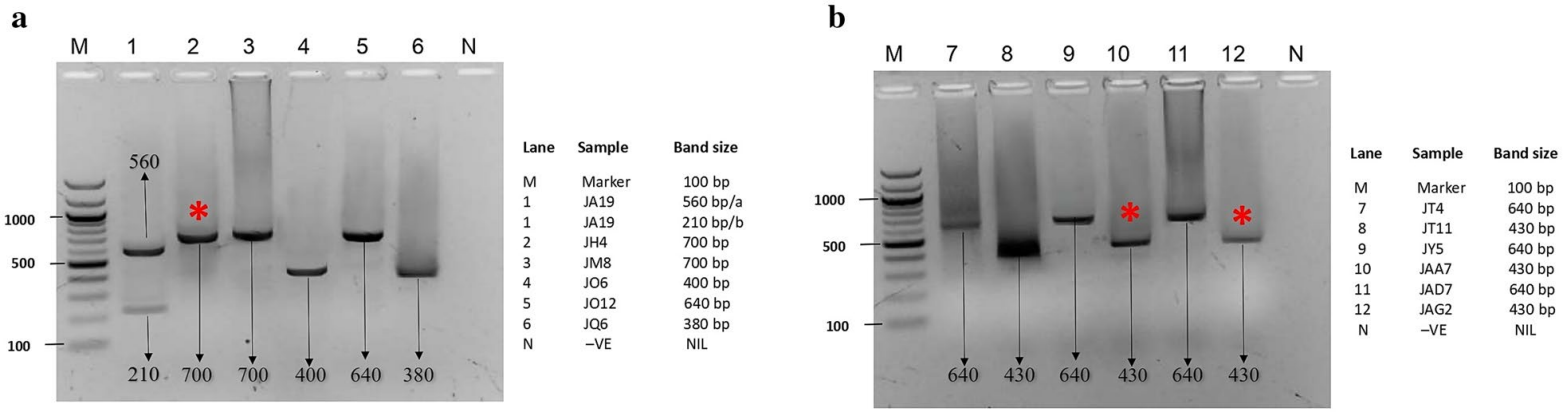
PCR amplification of trypanosome from blood of cattle using ITS-1 generic primers. **a** Lane 1 (JA19a and JA19b): A mixed infection with *T. congolense* Kilifi and *T. vivax*; lane 2 (JH4): *T. congolense* Savannah; lane 3 (JM8): *T. congolense* Savannah; lane 4 (JO6): *T. theileri*; lane 5 (JO12): *T. congolense* Savannah; **b** lane 6 (JQ6): *T. simiae*; lane 7 (JT4): *Tr. congolense* Savannah; lane 8 (JT11): *T. evansi*; lane 9 (JY5): *T. congolense* Savannah; lane 10 (JAA7): *Tr. brucei brucei*; lane 11 (JAD7): *T. congolense* Forest; lane 12 (JAG2): *T. brucei brucei*. *Positive for microscopy. Lanes M: 100 bp supperladder-mid (ABgene). Lanes N: negative control

Across the study area, 72 herd clusters were identified as a sample frame, from which 50% of the herds representing 36 clusters were balloted by simple random sampling. In all, 398 blood samples were obtained from across the study area, three of which were screened positive by microscopy, representing 0.8% prevalence (Table 1), while 12 samples representing 3.0% tested positive by nested PCR, with distinct band size characteristics of the species involved (Fig. 1). Details on the herd location and number of samples obtained from each herd are contained in Additional file 1: Table S1.

**Table 1:** Prevalence of trypanosome infection among cattle in Jebba by microscopic examination

Cluster	Total cluster	Sampling point	Herd size	Total examined	+Ve	−Ve	Prevalence (%)	Species
Positive	3	JH	183	11	1	10	9.1	T. c spp.
		JAA	172	10	1	9	10	T. b spp.
		JAG	92	6	1	5	16.7	T. b spp.
Negative	33	Others	6156	371	0	371	0	Nil
Total	36		6603	398	3	395	0.8	

*T. c spp* *Trypanosoma congolense* species,* T. b spp* *Trypanosoma brucei* species, *JH* Jebba, cluster H, *JAA* Jebba, cluster AA, *JAG* Jebba, cluster AG

**Fig. 2:**
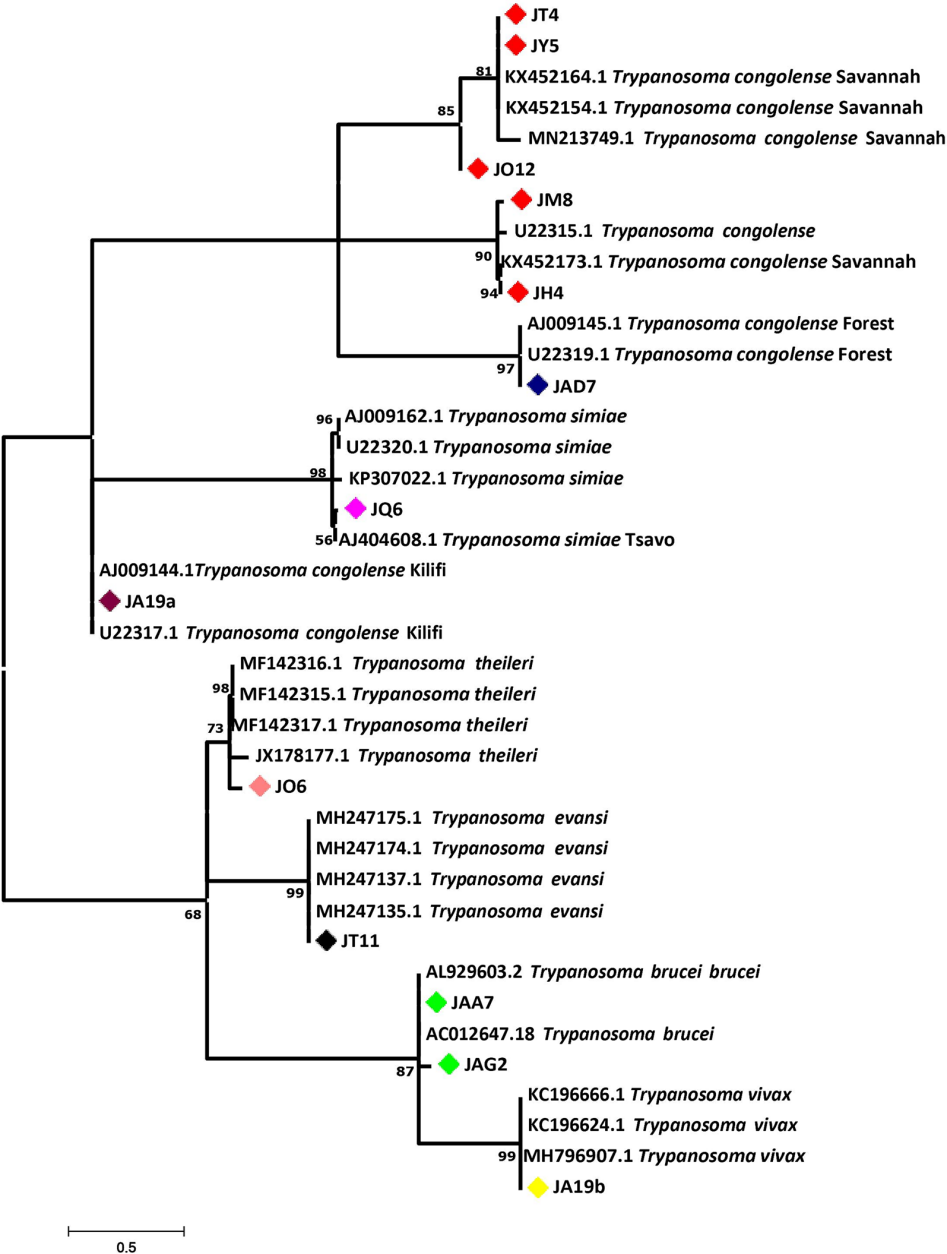
Phylogenetic relationships of trypanosomes within the subgenus *Nannomonas* clade deduced from small subunit rRNA gene sequences. The phylogram was constructed by bootstrapped (1000 replicates) maximum likelihood (ML) analysis based on the Tamura–Nei model. The tree with the highest log likelihood (−1113.90) is shown. Bootstrap values for all major nodes are given and all branches receiving bootstrap support values > 50%. The tree was drawn to scale, with branch lengths measured in the number of substitutions per site. The analysis involved 39 nucleotide sequences and 77 positions in the final dataset. Evolutionary analyses were conducted in MEGA7

Sequencing of the PCR products and bioinformatics analysis (http://blast.ncbi.nlm.nih.gov/Blast.cgi) further validated the species and subspecies of the trypanosomes (for details on analysed samples and sequences, see Additional file 3: Table S3 and Additional file 4: Dataset S1). To make an evolutionary inference, all isolates aligned with the salivarian group except JO6 (*T. theileri*), which fell within the stercorarian group. Small subunit RNA (ssrRNA) from JT11 (*T. evansi*), JAD7 (*T. brucei*) and JAG2 (*T. brucei*) fell within the same branch but different clades, which depicts a logical evolutionary event arising from within these species (Fig. 2). *Trypanosoma evansi* is widely known to have evolved from *T. brucei*, all of which were rooted on *T. vivax*.

Within the study area, four different breeds of cattle were found. Table 2 shows the effect of trypanosome infection on the PCV in each of the cattle breeds. For infected animals, PCV estimates followed the order Red Bororo < White Fulani < Sokoto Gudali, while the PCV for the non-infected animals followed the order White Fulani > Sokoto Gudali > Red Bororo > Muturu.

**Table 2:** Comparison of mean PCV among cattle breeds infected with *Trypanosoma* species in the study location (June 2019)

Cattle breed	No. of animals infected	Average haematocrit (%)	
		Infected (mean ± SEM)	Not infected (mean ± SEM)
Muturu	0	0	32.8 ± 1.23
Red Bororo	1	18.3 ± 0.84	34.2 ± 1.15
Sokoto Gudali	3	26.0 ± 1.82	35.0 ± 1.84
White Fulani	8	24.9 ± 1.70	35.7 ± 2.62

PCV values are means of three replicates of the same sample and are expressed as mean ± SEM

Different trypanosoma species were identified in cattle distributed within the study area as shown in Table 3. Comparatively, the PCV values for non-infected animals were higher than for those infected with individual parasites. The animals infected with *T. brucei* were the least affected, with an average PCV of 30.3 ± 0.92, while the animals infected with *T. theileri* were the most affected, with an average PCV of 19.2 ± 1.12. Generally, the PCV in regard to species infection followed the order *T. theileri* < *T. evansi* < *T. simiae* < *T. congolense* < *T. brucei* (Table 3). Details on the average PCV obtained for each herd and the trypanosome species and subspecies infections are contained in Additional file 2: Table S2, Additional file 5: Table S4 and Additional file 6: Table S5, respectively.

**Table 3:** Comparison of mean PCV of cattle infected with *Trypanosoma* species in the study location (June 2019)

S/No.	Species infection	Number of animals infected	PCV (%)
1	Non-infected	386	36.2 ± 3.71
2	*T. congolense*	7	23.8 ± 1.15
3	*T. brucei*	2	30.3 ± 0.92
4	*T. evansi*	1	20.0 ± 0.32
5	*T. theileri*	1	19.2 ± 0.12
6	*T. simiae*	1	22.0 ± 0.63

PCV values are means of three replicates of the same sample and are expressed as mean ± SEM

**Fig. 3:**
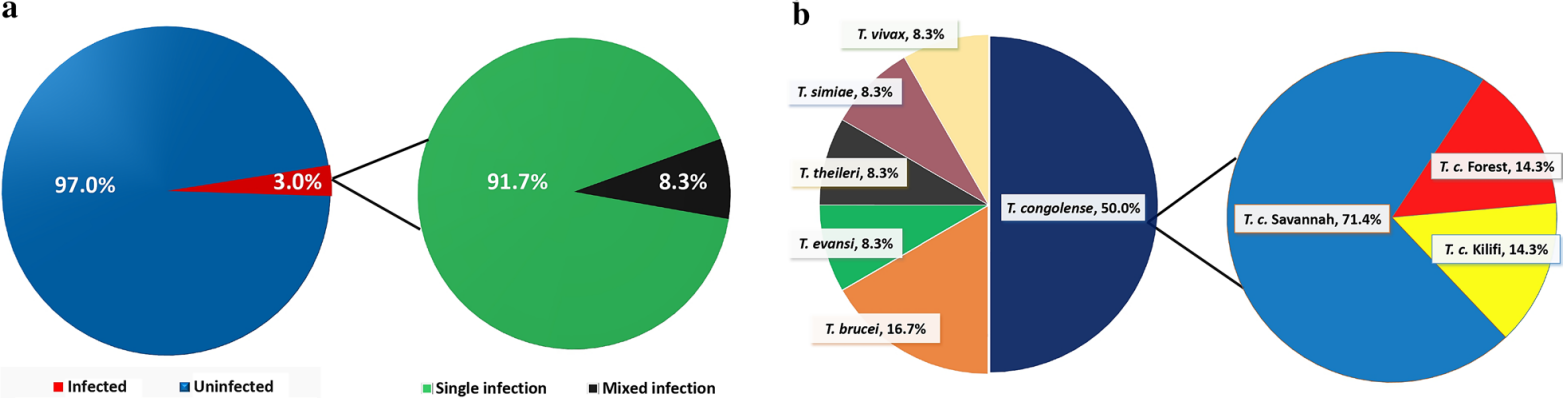
Prevalence of trypanosome infections among cattle distributed within the Jebba axis of the River Niger, Kwara state (June 2019). **a** Prevalence of single and mixed infections. **b** Prevalence according to species and subspecies infection

**Fig. 4:**
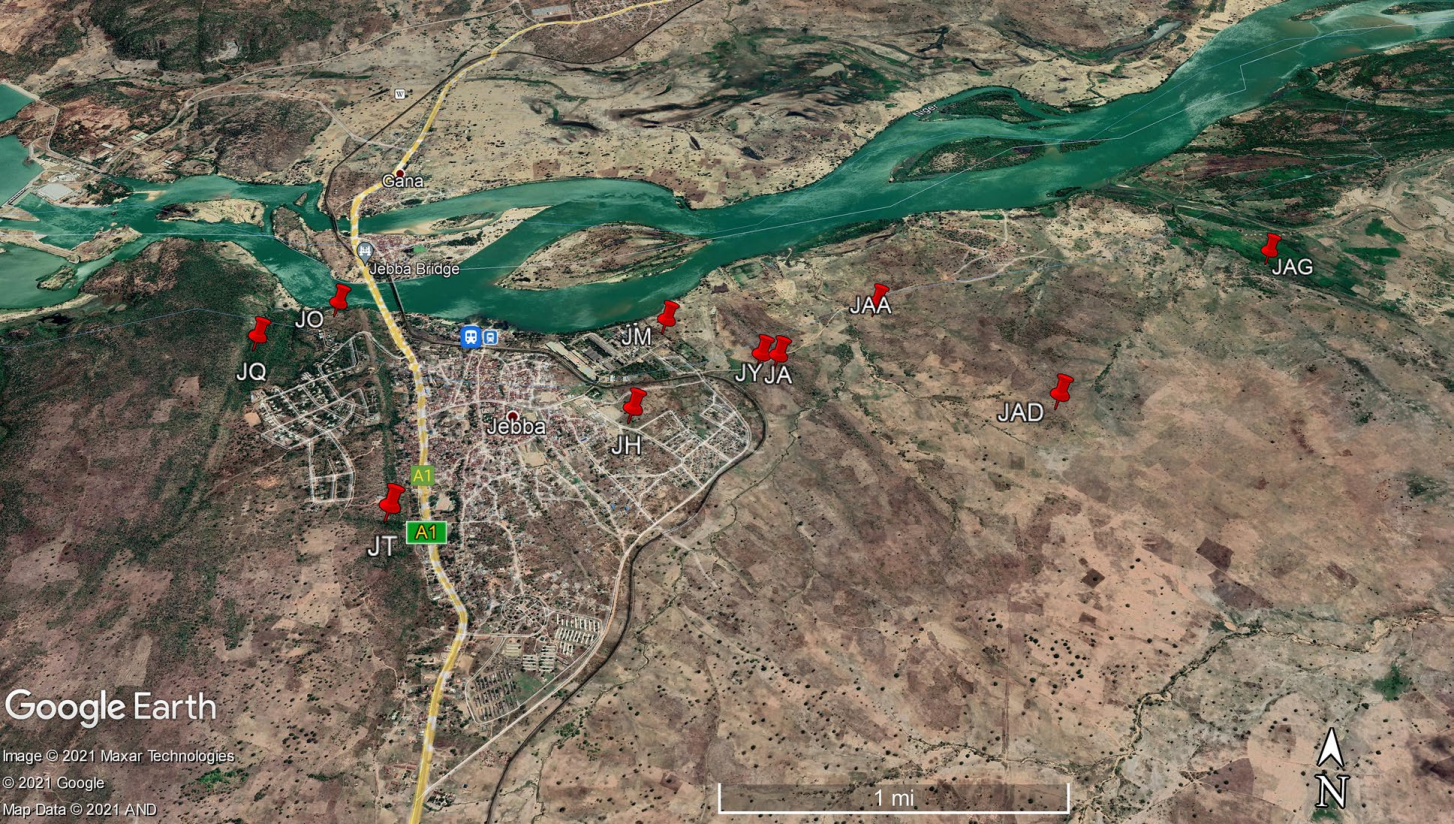
Spatial distribution of trypanosome-infected herds within the Jebba axis of the River Niger, Kwara state, Nigeria (June 2019)

 Trypanosome infection rates varied with sex, age, cattle breed, residency/stay period, animal origin, disease and treatment history, travel history, herd size and location of herd relative to the River Niger. As shown in Table 4, the infection rate was higher in female animals (4.3%), young animals (10.0%), White Fulani cattle breeds (3.7%), animals that were recently domiciled ≤ 3 years (3.2%), transhumance animals (3.6%) and animals having a disease history (4.1%). Similarly, in Table 5, the infection rate was higher among herds closer to the River Niger (56.2%), larger herds (33.3%) and animals that had travelled to endemic zones (50.0%). Aside from the age of animals and location distance from the River Niger, the differences in the infection rates for all tested parameters were not statistically significant. In Fig. 3a, our results show a single infection rate of 91.7% as against 8.3% recorded for mixed infection, with *T. congolense* being the most prevalent (50.0%), followed by *T. brucei brucei* (16.7%), while other species had equal prevalence (8.3%). In the study area, the infection rate with *T. congolense* Savannah type was comparatively higher (71.4%) than other types, as shown in Fig. 3b (see also Additional file 6: Table S5). The spatial distribution of trypanosome-infected herds across the study area is shown in Fig. 4.

**Table 4:** Parametric prevalence of trypanosome infection based on sex, age, breed, animal residency, origin, disease history and treatment history (June 2019)

Category	Subcategory	No. of cattle screened	No. of trypanosome infections	Prevalence (%)	*X*^2^	*P*-value
Sex	Male	165	2	1.2	3.133	0.077
	Female	233	10	4.3		
	Total	398	12	3.0		
Age (years)	≤ 1	70	7	10.0	14.172	0.000
	> 1	328	5	1.5		
	Total	398	12	3.0		
Breed	W.F.	214	8	3.7	1.172	0.760
	S.G.	130	3	2.3		
	R.B.	36	1	2.8		
	MUT	18	0	0.0		
	Total	398	12	3.0		
Animal residency (years)	≤ 3	126	4	3.2	0.016	0.899
	> 3	272	8	2.9		
	Total	398	12	3.0		
Animal origin	Sentinel	174	4	2.3	0.542	0.461
	Transhumance	224	8	3.6		
	Total	398	12	3.0		
Disease history	History	148	6	4.1	0.870	0.351
	No history	250	6	2.4		
	Total	398	12	3.0		
Treatment history	History	366	11	3.0	0.001	0.970
	No history	32	1	3.1		
	Total	398	12	3.0		

*WF *White Fulani, *RB* Red Bororo, *SK* Sokoto Gudali, *MUT* Muturu. χ^2^ = Chi-square test of association was carried out at a 95% confidence interval

**Table 5:** Parametric prevalence of trypanosome infection based on location, herd size and travel history to endemic zones (June 2019)

Category	Subcategory	No. of herds screened	No. of herd(s) infected	Prevalence (%)	*X*^2^	*P*-value
Location (Km) (distance from the River Niger	Far (> 3 km)	20	1	5.0	11.638	0.001
	Close (≤ 3 km)	16	9	56.2		
	Total	36	10	27.8		
Herd size	Large ≥ 200	15	5	33.3	0.396	0.529
	Small < 200	21	5	23.8		
	Total	36	10	27.8		
Travel history to endemic areas	Endemic	8	4	50.0	2.532	0.112
	Not endemic	28	6	21.4		
	Total	36	10	27.8		

Travel history: Kaduna, Ogun, Oyo, Jos, Benue and Delta. χ^2^ = Chi-square test of association was carried out at 95% confidence interval

## Discussion

The low prevalence reported in this study by microscopic screening (Table 1) is not surprising considering the generally low sensitivity of the parasitological diagnostic method [40–42]. This is especially so for field animals characterized by low parasitaemia. The superiority of PCR over the micro-haematocrit centrifugation technique (MHCT) has been widely demonstrated in epidemiological studies of animal trypanosomiasis [3, 32, 43, 44]. These differences are due to the sensitivity thresholds of the techniques. Nested PCR gave an overall prevalence of 3.0%. Each species tested produced amplicons of between 200 and 700 base pairs (bp) in length (Fig. 1a, b). The ITS-1 PCR product size of *T. evansi* was similar to that of *T. brucei*, and sequencing analysis was key to differentiating between the two PCR products. Sample JT11 was further confirmed to be *T. evansi* (Additional file 3: Table S3), suggesting the possible role of cattle as reservoirs of *T. evansi*. Generally, bands obtained from the amplification results agreed with those of previous studies [15, 22, 45–51]. Samples JH4 and JM8 resulted in band sizes of approximately 700 bp, which were confirmed by sequence analysis to be *T. congolense* Savannah type. Molecular characterization of *Trypanosoma* species using ITS-1 generic primers and/or their slight modification has been reported to give an estimated range of ITS-1 band sizes with a maximum amplicon length of 640 bp [15], and it was noted that all species amplification using generic primers could lead to a band size of between 150 and 750 bp in length, as evidenced in this study and those previously reported [29, 45, 50, 52]. An amplicon length of 210 bp for *T. vivax* was not reported previously, which may be an indication that *T. vivax* 18S rRNA is more rapidly evolving than that of other non-salivarian trypanosomes and also evolving significantly faster than all other trypanosomes [53]. Further biological characterization will depend on isolation of a living specimen into culture. The ability to identify this trypanosome by the distinct size of the ITS-1 region has provided preliminary information on its distribution that should help track it in the field.

A drastic decrease in PCV has traditionally been considered a warning sign of trypanosomiasis [54, 55]. Classically, infection with trypanosome species that are pathogenic in local breeds of cattle result in retarded growth and anaemia, and nutritional status is a determining factor of infection [56, 57]. From our findings, PCV was decreased in trypanosome-positive animals (Table 3; Additional file 5: Table S4), possibly due to the effect of parasites on blood cells. Similarly, the average haematocrit values varied between cattle breeds (Table 2). However, the very low PCV presented by the Red Bororo breed may not be a true reflection of the PCV trend, as only one animal was screened positive. Trypanosome species had different effects on the PCV status of animals, with the average falling below the standard obtainable for cattle (24–46%), except for the animals infected with *T. brucei*, with an average PCV of 30.3 ± 0.92. The animal infected with *T. theileri*, a non-pathogenic trypanosome of cattle, had the lowest PCV value (19.2 ± 0.12), but it is difficult to conclude that this decrease was actually caused by *T. theileri* (Table 3). Co-infections or other possible causes of decreased PCV cannot be ruled out. However, it is possible that the parasite may have transited from a non-pathogenic to pathogenic form, hence the need for a controlled experiment aimed at monitoring the PCV in the face of *T. theileri* infection and other trypanosome species.

This study showed 3.0% overall prevalence, which compares well with the 4.3% national prevalence as reported by the European Economic Commission project of 1989 and 1996 [58], 3.9% in Ogbomosho [59], 4.7% in Oyo [60] and 9.4% in Kaduna [61], as against high prevalence of 53.4% in Kaura [62] and 46.8% in Jos [63]. These findings might reflect seasonal or local differences in tsetse populations, sample size and site, improved sensitization among nomads on grazing courses, better implementation of the use of trypanocidal drugs and urbanization.

Our results showed that females were more commonly infected (Table 4). This observed difference may be attributed to livestock management practices adopted in the farming community, wherein larger numbers of males are frequently sold off the herd at an early age while the rest are kept for breeding or animal traction. Also, females persist longer in herds for the purpose of breeding, thus allowing the chronic infection to be maintained for very long period. As a result, the remaining males are more closely monitored while the females are readily exposed to hazard in the population vis à vis multiple copulation with limited male animals in the herd. Also, the larger population of females (59%) obtained in this study by simple random sampling may account for this difference. Previously, 199 males and 121 females were examined in the Ido Local Government Area (LGA), Oyo State, Nigeria, with no statistical difference in the infection prevalence [60]. Factors other than sex relating to the host or its environment could therefore have played a role in the susceptibility of animals to infection, which has been documented in several studies [8, 10, 64].

From our study, there is a decrease in disease prevalence as animals age, probably due to age-acquired immunity, which could represent a key positive factor, and bearing in mind that trypanocidal treatments are more frequently used on adults by local farmers. In addition, young animals are more vulnerable to tsetse bites due to their skin fragility. Moreover, they are not agile enough to ward off insects along the grazing route as are the adults. The tsetse flies also frequently target weak animals as a source of food in order to avoid being crushed by moving animals [65]. In this study, despite the very low number of young animals (17.6%) randomly sampled (Table 4), a prevalence of 10.0% (7/70) was recorded for younger animals and 1.5% (5/328) for adults, which is statistically significant (*χ*^2^ = 14.172, *df* = 1, *P* < 0.001). This indicates that the incidence rate was not similar between young and adult animals [8]. The prevalence of trypanosome infection was lowest in Sokoto Gudali (2.3%), a breed not known for being tolerant to trypanosome infection [66, 67]. The higher prevalence observed among the White Fulani breed may be attributed to their known susceptibility and perhaps due to their higher representation in the sampling (52.8%). Of the four cattle breeds studied, the White Fulani are usually raised under the nomadic system of management. This may be another possible explanation for the higher prevalence recorded in this cattle group [10].

The prevalence of trypanosome infection was higher in locations closer to the River Niger as compared to those further away (Table 5; Fig. 4). This difference is significant at a 95% confidence interval (*χ*^2^ = 11.638, *df* = 1, *P* < 0.05) and may be attributed to differences in herd management practices, grazing routes which predispose the herd to tsetse bites and herd composition which may differ in each herd. The river and the vegetation along its course could be a positive factor for the vector transmitting the disease as well as a source of water for grazing animals, which could expose them to risk of bite by riverine species of the flies [68].

The majority of the trypanosomes in cattle were *T. congolense* and *T. brucei*, with nearly half of the overall infection due to *T. congolense* Savannah type (Fig. 3b), possibly as a result of the large host range or likely due to the fact that riverine species of tsetse are generally considered susceptible to *T. congolense* infections [69]. The relatively low prevalence of *T. brucei* as compared to *T. congolense* may be associated with the poor efficiency of the generic ITS-1 primers in identifying members of the subgenus *Trypanozoon* (e.g., *T. brucei* subspecies) [70]. The high prevalence of *T. congolense* infection is an indication of the dominance of the *Glossina morsitans* tsetse fly [7, 8, 30, 32, 63, 71, 72] and could mean that its transmission is highly favoured by the obligate cyclical vector or that *T. vivax* and *T. brucei* respond better to the trypanocidal drugs, diminazene aceturate and homidium chloride, indiscriminately administered by farmers. A high prevalence of the Savannah subgroup in cattle may also indicate that the parasites were introduced recently into the tested herds, coupled with its reported virulence as compared to other subgroups [73]. The low prevalence of *T. brucei* infection may relate to the reported resistance of indigenous West African cattle to the parasite [74]. However, the detection of *T. brucei* and *T. evansi* in Nigerian cattle might portend serious danger not only to cattle and other livestock but also to livestock owners and the host communities at large, as *T. evansi* infection has been reported in cattle and humans [75, 76]. The low prevalence of *T. simiae* infection is an indication of low transmission of the parasite, as animals infected with this species will probably not survive the acute and severe nature of the effects of this parasite [77, 78]. Double infections in animals are a normal occurrence in the field [79]. This study identified *T. congolense* (Kilifi type) and *T. vivax* mixed infection in only one of the tested herds. In Nigeria, previous surveys identified mainly *T. congolense* and *T. vivax* as animal-pathogenic trypanosomes [80], and co-circulation has been reported in studies conducted in northern Nigeria [22, 32, 81]. Co-infections with multiple *Trypanosoma* species have also been documented previously due to bites from tsetse flies carrying more than one *Trypanosoma* infection or successive bites from flies with different *Trypanosoma* species [8, 82, 83].

## Conclusions

Six species of trypanosomes were identified in cattle herds along the Jebba axis of the River Niger, with *T. congolense* being the most prevalent. Age and relative distance of herds from the River Niger may be risk factors for trypanosome infection in cattle herds along the Jebba axis of the River Niger. According to the threshold of the European Economic Commission (i.e., 4.3%), the study area may not be classified as endemic, but our data suggest that the cattle population may play a role in the possible resurgence of the disease in this region.

## Supplementary Information

**Additional file 1: Table S1.** Cluster data of herds screened for *Trypanosoma* infection in Jebba, Kwara State, Nigeria.

**Additional file 2: Table S2.** Cluster prevalence of *Trypanosoma* infection and average PCV of cattle in Jebba, Kwara State, Nigeria (June 2019)

**Additional file 3: Table S3:** Sequence similarity of ITS-1 DNA sequences of trypanosomes isolated from cattle with other sequences available in GenBank.

**Additional file 4: Dataset S1:** The DNA sequence data of *Trypanosoma* species obtained by BigDye Terminator Cycle Sequencing Kits v3.1.

**Additional file 5: Table S4.** Prevalence of *Trypanosoma* species infection among cattle in Jebba, Kwara State, Nigeria (June 2019).

**Additional file 6: Table S5.** Prevalence of *Trypanosoma congolense* infection (according to types: Kilifi, Savannah, and Forest), among cattle in Jebba, Kwara State, Nigeria (June 2019)

## Data Availability

All data generated or analysed during this study are included in this published article and its supplementary data files.

## Abbreviations

AT: African trypanosomiasis
AAT: African animal trypanosomiasis
HAT: Human African trypanosomiasis
PCR: Polymerase chain reaction
ITS-1: Internally transcribed spacers-1
HCT: Haematocrit centrifugation technique
BCM: Buffy coat method
PCV: Packed cell volume

## References

[1] De Gier J, Cecchi G, Paone M, Dede P, Zhao W (2020). The continental Atlas of tsetse and African animal trypanosomosis in Nigeria. Acta Trop.

[2] Food and Agriculture Organisation of the United Nations, (FAO). Food, agriculture and food security: the global dimension, 2002. WFS02/Tech/Advanced Unedited Version. Rome p. 19–28.

[3] Food and Agriculture Organisation of the United Nations, (FAO). Food, agriculture and food security: trypanosomosis 2003. Available from: http://www.spc.int/rahs/. Accessed 27 Aug 2019.

[4] Shiferaw S, Muktar Y, Belina D (2015). A review on trypanocidal drug resistance in Ethiopia. J Parasitol Vec Biol.

[5] World Health Organization report on elimination of African trypanosomiasis (*Trypanosoma brucei gambiense*) Geneva, Switzerland 2012.

[6] World Health Organization. human African trypanosomiasis, (sleeping sickness) 2016. https://www.who.int/trypanosomiasis_african/en. Accessed 26 May 2019.

[7] Ravel S, Mediannikov O, Bossard G, Desquesnes M, Cuny G, Davoust B (2015). A study on African Animal trypanosomosis in four areas of Senegal. Folia Parasitol.

[8] Farougou S, Allou SD, Sankamaho I, Codjia V (2012). Prevalence of trypanosome infections in cattle and sheep in the Benin’s West Atacora agro-ecological zone. Tropicultura.

[9] Kyambadde JW, Enyaru JCK, Matovu E, Odiit M, Carasco JF (2000). Detection of trypanosomes in suspected sleeping sickness patients in Uganda using the polymerase chain reaction. Bull World Health Organization..

[10] Karshima SN, Lawal IA, Bata SI, Barde IJ, Adamu PV, Salihu A (2016). Animal reservoirs of *Trypanosoma brucei gambiense* around the old Gboko sleeping sickness focus in Nigeria. J Parasitol Vec Biol.

[11] Umeakuana PU, Gibson W, Ezeokonkwo RC, Anene BM (2019). Identification of *Trypanosoma brucei gambiense* in naturally infected dogs in Nigeria. Parasit Vectors.

[12] Truc P, Buscher P, Desquenes M (2013). Atypical human infections by animal trypanosomes. PLoS Negl Trop Dis.

[13] Truc P, Gibson W, Herder S (2007). Genetic characterization of *Trypanosoma evansi* isolated from a patient in India. Infect Gen Evol.

[14] Deborggraeve S, Koffi M, Jamonneau V, Bonsu FA, Queyson R (2008). Molecular analysis of archived blood slides reveals an atypical human *Trypanosoma* infection. Diagn Microbiol Infect Dis.

[15] Adams ER, Malele II, Msangi AR, Gibson WC (2006). Trypanosome identification in wild tsetse populations in Tanzania using generic primers to amplify the ribosomal RNA ITS-1 region. Acta Trop.

[16] Mulenga GM, Namangala B, Chilongo K, Mubamba C, Hayashida K, Henning L (2021). Challenges in the diagnostic performance of parasitological and molecular tests in the surveillance of African trypanosomiasis in eastern Zambia. Trop Med Infect Dis.

[17] Magez S, Pinto Torres JE, Oh S, Radwanska M (2021). Salivarian trypanosomes have adopted intricate host-pathogen interaction mechanisms that ensure survival in plain sight of the adaptive immune system. Pathogens.

[18] Traub RJ, Monis PT (2005). Robertson ID, Molecular epidemiology: a multidisciplinary approach to understanding parasitic zoonoses. Int J Parasitol.

[19] Geysen D, Delespaux V, Geerts S (2003). PCR-RFLP using Ssu-rDNA amplification as an easy method for species-specific diagnosis of *Trypanosoma* species in cattle. Vet Parasitol.

[20] Gibson WC, Dukes P, Gashumba JK (1988). Species-specific DNA probes for the identification of African trypanosomes in tsetse flies. Parasitology.

[21] Kukla BA, Majiwa PA, Young JR, Moloo SK (1987). Use of species-specific DNA probes for detection and identification of trypanosome infection in tsetse flies. Parasitology.

[22] Weber JS, Ngomtcho HSC, Shaida SS, Chechet GD, Gbem TT, Nok JA (2019). Genetic diversity of trypanosome species in tsetse flies (*Glossina* spp.) in Nigeria. Parasit Vectors.

[23] Ngomtcho SC, Weber JS, Bum EN, Gbem TT, Kelm S, Achukwi MD (2017). Molecular screening of tsetse flies and cattle reveal different *Trypanosoma* species including *T. grayi* and *T. theileri* in northern Cameroon. Parasit Vectors.

[24] Masiga DK, Smyth AJ, Hayes P, Bromidge TJ, Gibson WC (1992). Sensitive detection of trypanosomes in tsetse flies by DNA amplification. Int J Parasitol.

[25] Moser DR, Cook GA, Ochs DE, Bailey CP, McKane MR, Donelson JE (1989). Detection of *Trypanosoma congolense* and *Trypanosoma brucei* subspecies by DNA amplification using the polymerase chain reaction. Parasitology.

[26] McNamara JJ, Laveissiere C, Masiga DK (1995). Multiple trypanosome infections in wild tsetse in Côte d'Ivoire detected by PCR analysis and DNA probes. Acta Trop.

[27] Solano P, Argiro L, Reifenberg JM, Yao Y, Duvallet G (1995). Field application of the polymerase chain reaction (PCR) to the detection and characterization of trypanosomes in *Glossina longipalpis* (Diptera: Glossinidae) in Côte d'Ivoire. Mol Ecol.

[28] Woolhouse ME, McNamara JJ, Hargrove JW, Bealby KA (1996). Distribution and abundance of trypanosome (subgenus Nannomonas) infections of the tsetse fly *Glossina pallidipes* in southern Africa. Mol Ecol.

[29] Desquesnes M, McLaughlin G, Zoungrana A, Davila AMR (2001). Detection and identification of trypanosomes of African livestock through a single PCR based on internal transcribed spacer 1 of rDNA. Int J Parasitol.

[30] Njiru ZK, Makumi JN, Okoth S, Ndungu JM, Gibson WC (2004). Identification of trypanosomes in *Glossina pallidipes* and *G. longipennis* in Kenya. Infect Genet Evol.

[31] Oyebanji JO (1982). Quality of life in Kwara state, Nigeria: an exploratory geographical study. Soc Indic Res.

[32] Takeet IM, Fagbemi BO, De Donato M, Yakubu A, Rodulfo HE, Peters SO (2013). Molecular survey of trypanosomes in naturally infected Nigerian cattle. Res in Vet Sci.

[33] Thrusfield M (2007). Veterinary epidemiology.

[34] Ledoka MV. Molecular characterization of trypanosomes commonly found in cattle, wild animals and tsetse flies In Kwazulu. being a thesis submitted for an award of the master’s degree in the department of veterinary tropical disease, faculty of veterinary sciences, University of Pretoria; 2008.

[35] Herbert WJ, Lumsden WH (1976). *Trypanosoma brucei:* A rapid ‘‘matching’’ method for estimating the host’s parasitemia. Exp Parasitol.

[36] Gagman HA, Ajayi OO, Yusuf AS (2014). A survey for haemo-parasite of pigs slaughtered in jos abattoir Plateau State Nigeria. Bayero J Pure Appl Sci..

[37] Cheesbrough M. District laboratory practice in tropical countries, part 2. Press syndicate of the University of Cambridge UK; 2000. p. 320–321.

[38] Chandhri SS. Goupte SK. Manual of general veterinary parasitology. 1st edn. Int book dist. co; 2003. p. 19–48.

[39] Ng’ayo MO, Njiru ZK, Muluvi GM, Osir EO, Masiga DK (2005). Kinetoplastid biology and disease detection of trypanosomes in small ruminants and pigs in western Kenya: important reservoirs in the epidemiology of sleeping sickness. Kinetoplast Biol Dis..

[40] Picozzi K, Tilley A, Fèvre EM, Coleman PG, Magona JW, Odiit M (2002). The diagnosis of trypanosome infections: applications of novel technology for reducing disease risk. Afr J Biotech.

[41] Abenga JN, Enwezor FNC, Lawani FAG, Osue HO, Ikemereh ECD (2004). Trypanosome prevalence in cattle in Lere area in Kaduna State. Revue Elev Med Vet Pay Trops.

[42] Enwezor FNC, Samdi SM, Ijabor O, Abenga JN (2012). The prevalence of bovine trypanosomes in parts of Benue State, north-central Nigeria. J Vect Borne Dis.

[43] Herrera HM, Da’vila AMR, Norek A, Abreu UGP, Souza SS, Dandrea OS (2004). Enzootiology of *Trypanosoma evansi* in Pantanal, Brazil. Vet Parasitol.

[44] Ferna’ndez D, Gonza’lez-Baradat B, Eleizalde MC, Gonza’lez-Marcano E, Perrone T, Mendoza M (2009). *Trypanosoma evansi:* a comparison of PCR and parasitological diagnostic tests in experimentally infected mice. Exp Parasitol.

[45] Da’vila AM, Herrera HM, Schlebinger T, Souza SS, Traub-Cseko YM (2003). Using PCR for unraveling the cryptic epizootiology of livestock trypanosomosis in the Pantanal, Brazil. Vet Parasitol.

[46] Gonzales JL, Jones TW, Picozzi K, Cuellar RH (2003). Evaluation of a polymerase chain reaction assay for the diagnosis of bovine trypanosomiasis and epidemiological surveillance in Bolivia. Kinetoplast Biol of Dis.

[47] Gonzales JL, Chacon E, Miranda M, Loza A, Siles LM (2007). Bovine trypanosomosis in the Bolivian Pantanal. Vet Parasitol.

[48] Mekata H, Konnai S, Witola WH, Inoue N, Onuma M, Ohashi K (2009). Molecular detection of trypanosomes in cattle in South America and genetic diversity of *Trypanosoma evansi* based on expression site-associated gene 6. Infect Genet Evol.

[49] Ramı’rez-Iglesias JR, Eleizalde MC, Reyna-Bello A, Mendoza M (2017). Molecular diagnosis of cattle trypanosomes in Venezuela: evidences of *Trypanosoma evansi* and *Trypanosoma vivax* infections. J Parasitol Dis..

[50] Nakayima J, Nakao R, Alhassan A, Mahama C, Afakye K, Sugimoto C (2012). Molecular epidemiological studies on animal trypanosomiases in Ghana. Parasit Vectors.

[51] Garcıa HA, Garcıa ME, Perez G, Bethencourt A, Zerpa E, Perez H (2006). Trypanosomiasis in Venezuelan water buffaloes: association of packed cell volumes with seroprevalence and current trypanosome infection. Ann Trop Med Parasitol.

[52] Njiru ZK, Constantine CC, Guya S, Crowther J, Kiragu JM, Thompson RC (2005). The use of ITS-1 rDNA PCR in detecting pathogenic African trypanosomes. Parasitol Res.

[53] Stevens J, Rambaut A (2001). Evolutionary rate differences in trypanosomes. Infect Genet Evol.

[54] Esievo KAN, Saror DI, Ilemobade AA, Hallaway MH (1985). Variation in erythrocyte surface and free serum sialic acid concentration during experimental *Trypanosoma vivax* infection in cattle. Res Vet Sci.

[55] Doko-Allou S, Farougou S, Salifou S, Ehile E, Geerts S (2010). Dynamique des infections trypanosomiennes chez les bovins Borgou sur la ferme de l’Okpara au Bénin. Tropicultura.

[56] Holmes PH, Katunguka-Rwakishaya E, Bennison JJ, Wassink GJ, Parkins JJ (2000). Impact of nutrition of pathophysiology of bovine trypanosomiasis. Parasitol.

[57] Murray M, D’Ieteren GDM. Teale AJ. From Trypanotolerance. In: Maudlin I, Holmes PH, Miles MA, editors. The Trypanosomiasis. Wallingford: CABI Publishing; 2004. p. 48.

[58] Onyiah JA (1997). African animal trypanosomosis: an overview of the current status in Nigeria. Trop Vet.

[59] Ameen SA, Joshua RA, Adedeji OS, Raheem AK, Akingbade AA, Leigh OO (2008). Preliminary studies on prevalence of ruminant trypanosomosis in Ogbomoso area of Oyo State, Nigeria. Mid East J Sci Res.

[60] Fasanmi OG, Okoroafor UP, Nwufoh OC, Bukola-Oladele OM, Ajibola ES (2014). Survey for *Trypanosoma* species in cattle from three farms in Iddo Local Government Area, Oyo State. Sokoto J Vet Sci.

[61] Agu WE, Kalejaiye JO, Olatunde AO (1990). Prevalence of bovine trypanosomosis in some parts of Kaduna and Plateau State, Nigeria. Bull Anim Health Prod Afr.

[62] Maikaje DB. Some aspects of the epidemiology and drug sensitivity of bovine trypanosomiasis in Kaura LGA of Kaduna State. PhD Thesis, Ahmadu Bello University, Zaria, Nigeria; 1998.

[63] Majekodunmi AO, Fajinmi A, Dongkum C, Picozzi K, Thrusfield MV, Welburn SC (2013). A longitudinal survey of African animal trypanosomiasis in domestic cattle on the Jos Plateau, Nigeria: prevalence, distribution and risk factors. Parasit Vectors.

[64] Shah SR, Phulan MS, Memon MA, Rind R, Bhatti A (2004). Trypanosome infections in camels. Pakistan Vet J.

[65] Bengaly Z, Ganaba R, Sidibe I, Duvallet G (1998). Infections trypanosomiennes dans la zone Sud-soudanienne du Burkina Faso. Rev Elev Méd Vét Pays trop.

[66] Ogunsanmi AO, Ikede BO, Akpavie SO (2000). Effect of management, season, vegetation zones and breed on the prevalence of bovine trypanosomosis in Southwestern Nigeria. Israel J Vet Med.

[67] Talabi AO, Otesile EB, Joshua RA, Oladosu LA (2012). Clinical observations on three Nigerian Zebu cattle breeds following experimental *Trypanosoma congolense* infection. Bull Anim Health Prod Afr.

[68] Munangandu MH, Siamudaala V, Munyeme M, Nalubamba KSA (2012). Review of ecological factors associated with the epidemiology of wildlife trypanosomiasis in the Luangwa and Zambezi valley ecosystems of Zambia. Interdiscip Perspect Inf Dis..

[69] Reifenberg JM, Cuisance D, Frezil JL, Cuny G, Duvallet G (1997). Comparison of the susceptibility of different Glossina species to simple and mixed infections with *Trypanosoma* (Nannomonas) *congolense* savannah and riverine forest types. Med Vet Ent.

[70] Ahmed HA, Picozzi K, Welburn SC, MacLeod ET (2013). A comparative evaluation of PCR-based methods for species-specific determination of African Animal Trypanosomes in Ugandan cattle. Parasit Vectors.

[71] Peacock L, Ferris V, Bailey M, Gibson W (2012). The influence of sex and fly species on the development of trypanosomes in tsetse flies. PLoS Negl Trop Dis.

[72] Simukoko H, Marcotty T, Phiri I, Geysen D, Vercruysse J, Van den Bossche P (2007). The comparative role of cattle, goats and pigs in the epidemiology of livestock trypanosomiasis on the plateau of eastern Zambia. Vet Parasitol.

[73] Bengaly Z, Sidibe I, Ganaba R, Desquesnes M, Boly H, Sawadogo L (2002). Comparative pathogenicity of three genetically distinct types of *Trypanosoma congolense* in cattle: clinical observations and haematological changes. Vet Parasitol.

[74] Kalu AU (1996). Current status of tsetse fly and animal trypanosomosis on the Jos Plateau, Nigeria. Prev Vet Med.

[75] Laha RE, Sasmal NK (2009). Detection of *T. evansi* infection in clinically ill cattle, buffaloes and horses using various diagnostic tests. Epidemiol Inf.

[76] Joshi PP, Shegokar VR, Powar RM, Herder S, Katti R, Salkar HR (2005). Human trypanosomiasis caused by *Trypanosoma evansi* in India: the first case report. Am J Trop Med and Hyg.

[77] Sidibe I, Bengaly Z, Boly H, Ganaba R, Desquesnes M, Sawadogo L (2002). Differential pathogenicity of *Trypanosoma congolense* subgroup: implication for the strategic control of trypanosomiasis. Newsl Integr Contr Pathogenic Trypanosomes Vect (ICPTV).

[78] Simo G, Asonganyi T, Nkinin SW, Njiokou F, Herde S (2006). High prevalence of group 1 in pigs from the fontem sleeping sickness focus in Cameroon. Vet Parasitol.

[79] Mugittu KN, Silayo RS, Majiwa PAO, Kimbita EK, Mutayoba BM, Maselle R (2000). Application of PCR and DNA probes in the characterization of trypanosomes in the blood of cattle in farms in Morogoro Tanzania. Vet Parasitol.

[80] Odeniran PO, Ademola IO (2018). Meta-analysis of the prevalence of African Animal Trypanosomiasis in Nigeria from 1960 to 2017. Parasit Vectors.

[81] Isaac C, Ciosi M, Hamilton A, Scullion KM, Dede P, Igbinosa IB (2016). Molecular identification of different trypanosome species and subspecies in tsetse flies of northern Nigeria. Parasit Vectors.

[82] Malele I, Craske L, Knight C, Ferris V, Njiru Z (2003). The use of specific and generic primers to identify trypanosome infections of wild tsetse flies in Tanzania by PCR. Infect Genet Evol.

[83] Mekata H, Konnai S, Simuunza M, Chembensofu M, Kano R, Witola WH (2008). Prevalence and source of trypanosome infections in field-captured vector flies (*Glossina pallidipes*) in Southeastern Zambia. J Vet Med and Sci.

