# Supplementary material for: Molecular identification and prevalence of trypanosomes in cattle distributed within the Jebba axis of the River Niger, Kwara state, Nigeria
Source: Parasit Vectors. 2021 Oct 29;14:560. doi: 10.1186/s13071-021-05054-0 (PMC8557008; doi:10.1186/s13071-021-05054-0)
Supplement: Supplementary file 1 — Additional file 1: Table S1. Cluster data of herds screened for Trypanosoma infection in Jebba, Kwara State, Nigeria. [file 13071_2021_5054_MOESM1_ESM.docx]

**Table S1: Cluster data of herds screened for *Trypanosoma* infection in Jebba, Kwara State, Nigeria.**

| **CLUSTER** | **COORDINATE** | **DISTANCE FROM RIVER NIGER (Km)** | **HERD SIZE** | **PROPORTION TO HERD SIZE (6%)** | **MICROSCOPY** | **SPECIE** |
| --- | --- | --- | --- | --- | --- | --- |
| JA | 9°07'28.7"N 4°50'23.3"E | 2.270 | 350 | 21 | **-** | - |
| JB | 9°06'35.6"N 4°49'49.1"E | 3.840 | 233 | 14 | **-** | - |
| JC | 9°07'27.6"N 4°50'33.4"E | 2.500 | 267 | 16 | **-** | - |
| JD | 9°06'44.9"N 4°50'06.6"E | 3.710 | 200 | 12 | **-** | - |
| JE | 9°06'44.2"N 4°49'43.9"E | 3.310 | 271 | 16 | **-** | - |
| JF | 9°06'36.5"N 4°49'49.4"E | 3.730 | 150 | 09 | **-** | - |
| JG | 9°07'35.6"N 4°48'33.1"E | 0.995 | 117 | 07 | **-** | - |
| JH | 9°07'12.0"N 4°49'58.1"E | 1.880 | 183 | 11 | **+** | T. c |
| JI | 9°06'32.4"N 4°49'53.2"E | 4.120 | 167 | 10 | **-** | - |
| JJ | 9°06'44.9"N 4°49'17.7"E | 3.400 | 306 | 18 | **-** | - |
| JK | 9°06'34.9"N 4°49'45.1"E | 3.910 | 152 | 09 | **-** | - |
| JL | 9°06'51.8"N 4°49'39.1"E | 2.760 | 228 | 14 | **-** | - |
| JM | 9°07'39.4"N 4°50'02.8"E | 0.449 | 238 | 14 | **-** | - |
| JN | 9°07'35.4"N 4°49'34.2"E | 0.178 | 139 | 08 | **-** | - |
| JO | 9°07'42.0"N 4°48'51.3"E | 0.185 | 229 | 14 | **-** | - |
| JP | 9°07'34.0"N 4°48'41.6"E | 0.716 | 218 | 13 | **-** | - |
| JQ | 9°07'27.3"N 4°48'38.9"E | 1.100 | 176 | 11 | **-** | - |
| JR | 9°06'32.0"N 4°49'10.5"E | 4.280 | 157 | 09 | **-** | - |
| JS | 9°06'49.4"N 4°49'14.7"E | 3.190 | 257 | 15 | **-** | - |
| JT | 9°06'43.3"N 4°49'16.6"E | 3.380 | 237 | 14 | **-** | - |
| JU | 9°06'16.1"N 4°48'49.5"E | 5.370 | 171 | 10 | **-** | - |
| JV | 9°05'59.3"N 4°49'04.5"E | 6.210 | 212 | 13 | **-** | - |
| JW | 9°06'37.6"N 4°50'15.7"E | 4.270 | 123 | 07 | **-** | - |
| JX | 9°05'58.3"N 4°49'57.9"E | 6.020 | 091 | 06 | **-** | - |
| JY | 9°07'28.4"N 4°50'26.8"E | 1.290 | 162 | 10 | **-** | - |
| JZ | 9°07'56.5"N 4°50'39.5"E | 1.190 | 194 | 12 | **-** | - |
| JAA | 9°07'47.1"N 4°50'48.1"E | 1.960 | 172 | 10 | **+** | T. b |
| JAB | 9°07'45.5"N 4°51'02.4"E | 2.210 | 118 | 07 | **-** | - |
| JAC | 9°07'14.9"N 4°50'59.6"E | 4.090 | 129 | 08 | **-** | - |
| JAD | 9°07'18.6"N 4°51'23.2"E | 2.410 | 206 | 12 | **-** | - |
| JAE | 9°07'18.0"N 4°51'34.9"E | 3.860 | 215 | 13 | **-** | - |
| JAF | 9°07'32.5"N 4°52'21.2"E | 5.400 | 084 | 05 | **-** | - |
| JAG | 9°08'09.6"N 4°52'19.1"E | 2.920 | 092 | 06 | **+** | T. b |
| JAH | 9°08'15.1"N 4°52'36.6"E | 3.280 | 143 | 09 | **-** | - |
| JAI | 9°07'44.2"N 4°52'42.5"E | 5.570 | 125 | 08 | **-** | - |
| JAJ | 9°07'50.8"N 4°52'36.9"E | 5.210 | 108 | 07 | **-** | - |
| 36 |  |  | 6603 | 398 | 3 | 3 |

T. c: *Trypanosoma congolense* specie, T. b: *Trypanosoma brucei* specie, N: North, E: East
