# Supplementary material for: Molecular identification and prevalence of trypanosomes in cattle distributed within the Jebba axis of the River Niger, Kwara state, Nigeria
Source: Parasit Vectors. 2021 Oct 29;14:560. doi: 10.1186/s13071-021-05054-0 (PMC8557008; doi:10.1186/s13071-021-05054-0)
Supplement: Supplementary file 2 — Additional file 2: Table S2. Cluster prevalence of Trypanosoma infection and average PCV of cattle in Jebba, Kwara State, Nigeria (June 2019) [file 13071_2021_5054_MOESM2_ESM.docx]

| JA |  | 21 | 37.9±1.24 | 1 | 4.8 |
| --- | --- | --- | --- | --- | --- |
| JB |  | 14 | 37.7±1.11 | 0 | 0 |
| JC |  | 16 | 36.1±1.59 | 0 | 0 |
| JD |  | 12 | 33.9±1.39 | 0 | 0 |
| JE |  | 16 | 34.5±1.30 | 0 | 0 |
| JF |  | 9 | 32.6±1.55 | 0 | 0 |
| JG |  | 7 | 33.7±2.01 | 0 | 0 |
| JH |  | 11 | 36.1±1.69 | 1 | 9.1 |
| JI |  | 10 | 39.6±1.28 | 0 | 0 |
| JJ |  | 18 | 34.8±2.00 | 0 | 0 |
| JK |  | 9 | 34.6±2.08 | 0 | 0 |
| JL |  | 14 | 36.3±1.22 | 0 | 0 |
| JM |  | 14 | 36.3±1.32 | 1 | 7.1 |
| JN |  | 8 | 36.8±1.49 | 0 | 0 |
| JO |  | 14 | 35.0±1.31 | 2 | 14.3 |
| JP |  | 13 | 35.5±1.04 | 0 | 0 |
| JQ |  | 11 | 34.7±1.76 | 1 | 9.1 |
| JR |  | 9 | 36.2±1.58 | 0 | 0 |
| JS |  | 15 | 35.1±1.35 | 0 | 0 |
| JT |  | 14 | 31.6±1.86 | 2 | 14.3 |
| JU |  | 10 | 33.1±1.22 | 0 | 0 |
| JV |  | 13 | 36.5±1.53 | 0 | 0 |
| JW |  | 7 | 34.4±2.09 | 0 | 0 |
| JX |  | 6 | 32.0±2.48 | 0 | 0 |
| JY |  | 10 | 34.2±1.32 | 1 | 10 |
| JZ |  | 12 | 35.7±1.00 | 0 | 0 |
| JAA |  | 10 | 35.7±2.13 | 1 | 10 |
| JAB |  | 7 | 28.9±0.99 | 0 | 0 |
| JAC |  | 8 | 33.1±2.19 | 0 | 0 |
| JAD |  | 12 | 33.2±2.16 | 1 | 8.3 |
| JAE |  | 13 | 37.2±1.43 | 0 | 0 |
| JAF |  | 5 | 30.4±1.63 | 0 | 0 |
| JAG |  | 6 | 32.5±1.78 | 1 | 16.7 |
| JAH |  | 9 | 33.9±1.74 | 0 | 0 |
| JAI |  | 8 | 33.4±1.51 | 0 | 0 |
| JAJ |  | 6 | 34.0±2.22  TOTAL 398 34.6±0.36 12 3.02 | 0 | 0 |

| Cluster | No. of Animal  Sampled | Average PCV (%) | No. of animal  positive | Prevalence (%) |
| --- | --- | --- | --- | --- |

**Table S2: Cluster prevalence of *Trypanosoma* infection and average PCV of cattle in Jebba, Kwara State, Nigeria (June 2019)**
