# Supplementary material for: Molecular identification and prevalence of trypanosomes in cattle distributed within the Jebba axis of the River Niger, Kwara state, Nigeria
Source: Parasit Vectors. 2021 Oct 29;14:560. doi: 10.1186/s13071-021-05054-0 (PMC8557008; doi:10.1186/s13071-021-05054-0)
Supplement: Supplementary file 3 — Additional file 3: Table S3: Sequence similarity of ITS-1 DNA sequences of trypanosomes isolated from cattle with other sequences available in GenBank. [file 13071_2021_5054_MOESM3_ESM.docx]

**Table S3: Sequence similarity of ITS-1 DNA sequences of trypanosomes isolated from cattle with other sequences available in GenBank.**

| **S/No** | **Positive Sample** | **Approximate Band Size (bp)** | **BLAST % Similarity** | **E-Value** | **Genus/Specie Hit** | **Gene bank Accession No.** |
| --- | --- | --- | --- | --- | --- | --- |
| 1 | JA19a | 560 | 99.27 | 2e-61 | *T.c.* kilifi | [AJ009144.1](https://www.ncbi.nlm.nih.gov/nucleotide/AJ009144.1?report=genbank&log$=nucltop&blast_rank=1&RID=WRUVKHJG014) |
| 2 | JA19b | 210 | 99.32 | 5e-67 | *T. vivax* | [KC196666.1](https://www.ncbi.nlm.nih.gov/nucleotide/KC196666.1?report=genbank&log$=nucltop&blast_rank=1&RID=WRXDHEKK014) |
| 3 | JH4 | 700 | 90.29 | 1e-66 | *T.c.* Savannah | [MN213749.1](https://www.ncbi.nlm.nih.gov/nucleotide/MN213749.1?report=genbank&log$=nucltop&blast_rank=1&RID=WRV5A9CG014) |
| 4 | JM8 | 700 | 93.29 | 1e-52 | *T. c.* Savannah | [LC492129.1](https://www.ncbi.nlm.nih.gov/nucleotide/LC492129.1?report=genbank&log$=nucltop&blast_rank=4&RID=4DFKDHSM014) |
| 5 | JO6 | 400 | 88.48 | 1e-74 | *T. theileri* | [MF142319.1](https://www.ncbi.nlm.nih.gov/nucleotide/MF142319.1?report=genbank&log$=nucltop&blast_rank=2&RID=WRU19GZJ014) |
| 6 | JO12 | 640 | 87.19 | 2e-55 | *T. c.* Savannah | [MN213749.1](https://www.ncbi.nlm.nih.gov/nucleotide/MN213749.1?report=genbank&log$=nucltop&blast_rank=1&RID=WRVW612H015) |
| 7 | JQ6 | 380 | 90.41 | 4e-116 | *T. simiae* | [AJ404608.1](https://www.ncbi.nlm.nih.gov/nucleotide/AJ404608.1?report=genbank&log$=nucltop&blast_rank=1&RID=WRUKH4N0014) |
| 8 | JT4 | 640 | 86.28 | 2e-75 | *T. c.* Savannah | [MN213749.1](https://www.ncbi.nlm.nih.gov/nucleotide/MN213749.1?report=genbank&log$=nucltop&blast_rank=1&RID=WRW425PT014) |
| 9 | JT11 | 430 | 97.63 | 0.0 | *T. evansi* | [MH247175.1](https://www.ncbi.nlm.nih.gov/nucleotide/MH247175.1?report=genbank&log$=nucltop&blast_rank=1&RID=WRUBZEXD015) |
| 10 | JY5 | 640 | 86.64 | 4e-77 | *T. c.* Savannah | [MN213749.1](https://www.ncbi.nlm.nih.gov/nucleotide/MN213749.1?report=genbank&log$=nucltop&blast_rank=1&RID=WRVW612H015) |
| 11 | JAA7 | 430 | 95.04 | 3e-117 | *T. brucei brucei* | [AL929603.2](https://www.ncbi.nlm.nih.gov/nucleotide/AL929603.2?report=genbank&log$=nucltop&blast_rank=3&RID=WRWMWAX0014) |
| 12 | JAD7 | 640 | 95.78 | 2e-166 | *T. c.* Forest | [AJ009145.1](https://www.ncbi.nlm.nih.gov/nucleotide/AJ009145.1?report=genbank&log$=nucltop&blast_rank=1&RID=WRXRS2F1014) |
| 13 | JAG2 | 430 | 89.75 | 1e-91 | *T. brucei brucei* | [AL929603.2](https://www.ncbi.nlm.nih.gov/nucleotide/AL929603.2?report=genbank&log$=nucltop&blast_rank=3&RID=WRX2V1BF014) |

E-values as recorded are not in reference to each other but with respect to BLAST hit as presented on the NCBI database.
