# Supplementary material for: Molecular identification and prevalence of trypanosomes in cattle distributed within the Jebba axis of the River Niger, Kwara state, Nigeria
Source: Parasit Vectors. 2021 Oct 29;14:560. doi: 10.1186/s13071-021-05054-0 (PMC8557008; doi:10.1186/s13071-021-05054-0)
Supplement: Supplementary file 4 — Additional file 4: Dataset S1: The DNA sequence data of Trypanosoma species obtained by BigDye Terminator Cycle Sequencing Kits v3.1. [file 13071_2021_5054_MOESM4_ESM.docx]

**Dataset 1: The DNA sequence data of *Trypanosoma* species obtained by BigDye Terminator Cycle Sequencing Kits v3.1**

>JAA7

CAGTATGACTTTTACTTCTATATGCCGTTTGACATGGGTTCTAATATGTATGTGTGTATCTAAACTTATATTGTCCACATGGACATGGACATGGACTGGTACCCAATGGTATATATCCCACAACGTGTCGCGATGGATGACTTGGCTTCCTATTTCGTTGAAGAACGCAGCAAAGTGCGATAAGTGGTATCAATTGCAGAATCATTTCATTGATAATAGCCTGTAGGTACCTTTGAACGCAAACGGCGCATGGGAGAAGCTCTCTCGGAGCCATCCCCGTGCATGCCACATTTCTCAGTGTCGAATATAAAAACAAAACACACACCTATTTTTTGTGTTGTCTTGGTACTGCTTCCC

>JO12

CAGTAAAACACACCTGCGTACACACACAAGCACTCACCATATTTCCCCCACTGCACCTTCTTCCTCCAAACACACACTCTTCCTCCTCCTCTCTCCCAACACACGTGGGAAAGCGGAGAAAAGATGAGGATGAGGAGAAAAAAAGGGGAAAAAAAGGGCCCCGGGGCCCCCAAAAAAAGGCGGGGGGGGTAAAAAAAACCCTTCTCCCCCCCCCCCCCCCCTTTTTTTTCCTTTGGGGGGGGCACAAAATTAAAAACCCCCTCCACAAAAAAAAAAAAAAAAAACATGGGGGGCCCGTTTTTTGAAACCCGGGTTTTTCCAGGGGGTTTTTTAAAATTGGGGGGGGTAAAACAAAAACCCCCCCCCCCGGGGGGGTCCCCCAACCCCACCCCCCCCCCCCCGGGGGGGGGTGCGGGGGGAAATATTTTCCTTGAGGGGG

>JM8

CAGTAAAACACACCTGCGTACACACACAAGCACTCACCATATTTCCCCCACTGCACCTTCTTCCTCCAAACACACACTCTTCCTCCTCCTCTCTCCCAACACACGTGGGAAAGCGGAGAAAAGATGAGGATGAGGAGAAAAAAAGGGGAAAAAAAGGGCCCCGGGGCCCCCAAAAAAAGGCGGGGGGGGTAAAAAAAACCCTTCTCCCCCCCCCCCCCCCCTTTTTTTTCCTTTGGGGGGGGCACAAAATTAAAAACCCCCTCCACAAAAAAAAAAAAAAAAAACATGGGGGGCCCGTTTTTTGAAACCCGGGTTTTTCCAGGGGGTTTTTTAAAATTGGGGGGGGTAAAACAAAAACCCCCCCCCCCGGGGGGGTCCCCCAACCCCACCCCCCCCCCCCCGGGGGGGGGTGCGGGGGGAAATATTTTCCTTGAGGGGG

>JQ6

TATGACTTTTACTTGTGACTAAAATCGCTAGACCAAAGCAAAGCAGTCTAGCGACTTGAATTACCAAGCATGGGATAACAAAGCATCAGCCCCAGGGCCACCACCACCGTTTCGGCTTTGGATGGATGGATGGTTTTAGAAGTCCAGGGGAGATTATGGCGCCGCGTGCCGCGCCACACACCGTGTGCGTTTCGGCGTTCGCGCCGACGCGTGGTGCCAGGAATGCACGAGGGTAGTTCGGGGGAGAACTACTGGCGCGGTCAGAGGTCGAATTCTTAGACCGCGCCAAGACGAACTACAGCGAAGGCATTCCTTCAAGGATACCTTCCTACAATCAAGAACCAAGAAATTCCC

>JA19b

GAGTGACTGCAGCTGGATCATTTTCCGACCCTCTTCTCTTCTCTTCTCGTCGCGCCCGTCTCCCGGCCACCGGGGCGGGACAGCAAACCACGCAGCTGCCGCTCGACCGCGCCCCGCGCGCAGGTGGAGCACGGCCCACACAACGTGTCGCGATGGATGACTTGGCTTA

>JY5

CAGTAAAACACACCTGCGTACACACACAAGCACTCACCATATTTCCCCCACTGCACCTTCTTCCTCCAAACACACACTCTTCCTCCTCCTCTCTCCCAACACACGTGGGAAAGCGGAGAAAAGATGAGGATGAGGAGAAAAAAAGGGGAAAAAAAGGGCCCCGGGGCCCCCAAAAAAAGGCGGGGGGGGTAAAAAAAACCCTTCTCCCCCCCCCCCCCCCCTTTTTTTTCCTTTGGGGGGGGCACAAAATTAAAAACCCCCTCCACAAAAAAAAAAAAAAAAAACATGGGGGGCCCGTTTTTTGAAACCCGGGTTTTTCCAGGGGGTTTTTTAAAATTGGGGGGGGTAAAACAAAAACCCCCCCCCCCGGGGGGGTCCCCCAACCCCACCCCCCCCCCCCCGGGGGGGGGTGCGGGGGGAAATATTTTCCTTGAGGGGG

>JT4

CAGTAAAACACACATGCGTACACACACAAGCACTCACCATATTTCCCCCACTGCACCTTCTTCCTCCAAACACACACTCTTCCTCCTCCTCTCTCCCAACACACGTGGGAAAGCGGAGAAAAGATGAGGATGAGGAGAAAAAAAGGGGAAAAAAAGGGCCCCGGGGCCCCCAAAAAAAGGCGGGGGGGGTAAAAAAAACCCTTCTCCCCCCCCCCCCCCCCTTTTTTTTCCTTTGGGGGGGGCACAAAATTAAAAACCCCCTCCACAAAAAAAAAAAAAAAAAACATGGGGGGCCCGTTTTTTGAAACCCGGGTTTTTCCAGGGGGTTTTTTAAAATTGGGGGGGGTAAAACAAAAACCCCCCCCCCCGGGGGGGTCCCCCAACCCCACCCCCCCCCCCCCGGGGGGGGGTGCGGGGGGAAATATTTTCCG

>JA19a

GTCATGAGTGGAAAAAAAGGGGTCAAAAATTGGGTGTGGGGATATATTATATAGGGATATAGGGCGGCGCGCGCGGCCTCTCTCGAGGGAAACAAGAAACACGGGAGCGGTCCCCCACCCATTTTTTCTTACGCATGTCATGCATGCGGGGGCGTCCGTGATTTTTACTGTGACTAAAAAAGTGCGACCAAAGCAGTCCGCCGACTTGAATTACAAAGCATGACAGCGATTCACGACGCAGGCACGCCATGAATTGCTTACTGCTAAAAGCGGTTGCGTGCGTGGCGGCCCCAGCGTGTTGTTTGGTGCCCCAGGAGGAGGGCCGCCTTTACTACTGCCACGCTATGCTATGTGTAGTAAATAAGAGGTCCCAGCGGCATGGCACGTGTGTGTGGGTCGTGTGCGAGCCGGACATGTGATAGGATATAGAGATAAGGGTTCTTCTCTCTCGGGGCGCGCGCTCACGACGCAAGTGTGGCGCGTTGCTTTGGGGGACGGAATGGCACCACAGRTGCGTCAGAGGTGYAATTCTTANACCGRACCNANACRAACTACAGRGAYGGCATTTMTCSAGAG

>JT11

CGGAGTGACTGCAGCTGGATCATTTTCTGATATCCATTATACAAAAAAGAGCATATTTATGTGCATGTATAAATTGCACAGTATGCAACCAAAAATATACATATATGTTTTACATGTATGTGTTTCTATATGCCGTTTGACATGGGAGATGAGGGATGCTATACATAGTTCTGTTATTTTCTATCATGTATGTGTGTTAGAGTGTCTGTGTTAATATACTTTTTAATGCATGCTCTACATAATATACAGTAGTAATAACACAGAGAATACGTATGGAATGCGTATCTCTCTATATATATTTATGTATATATGCTATGTGTATATCAACCTCGCATATTTTCTCCCTGTGGACCCCACCTCCCCCACCGCGTGTCGATGGATGACTTGTGTTTTATA

>JO6

GTCATCACAAGGTATAAATCATCAAACTGAGCCGATTACGTCCCTGCCATTTTTTCTCACCGCCCGTCGTTGTTTCCGATGATGGTGCAATACACGCGAACGGACCGTCGATCGAATCGGTTGACCGAAAGTTCACCGATCTTGCTTCATACAGGAAGCAAAAATCGTAACCCCCCCGCTGTAGGTGAACCTGCAGCTGGATCATTTTCCGATGACATAAAGAATCACACATATGTTTATATGTACCGCGTCGTGGAATACAATTTTTTTTCTATTTTTCCACCTTCATACAGATATTTTTTTTATTGCAATATGTCTGTTGGTGGGGGGTGGGGGTTTACCCCCACCCCGACCCCAAACAAAGGGGGGGGTGAAAAATAGCGGGGCCCGATTCGCCCCCTTTTTGATGCGTGCCCCACAAAAAATTTCCCACAAGGGCCCCCCAACATTGGCAGAAAGGGAATTGGTTTTATATATATATGTGGGGATCTGTCATGTGAGTATGTGAAGCTCTGCTTTTTTCACACTTTTCTCCGCGCGCGACAAAAATTTGCACGAGAGAGACTTGGTTTTTAT

>JH4

CAGTAAAACACACCTGCGTACACACACAAGCACTCACCATATTTCCCCCACTGCACCTTCTTCCTCCAAACACACACTCTTCCTCCTCCTCTCTCCCAACACACGTGGGAAAGCGGAGAAAAGATGAGGATGAGGAGAAAAAAAGGGGAAAAAAAGGGCCCCGGGGCCCCCAAAAAAAGGCGGGGGGGGTAAAAAAAACCCTTCTCCCCCCCCCCCCCCCCTTTTTTTTCCTTTGGGGGGGGCACAAAATTAAAAACCCCCTCCACAAAAAAAAAAAAAAAAAACATGGGGGGCCCGTTTTTTGAAACCCGGGTTTTTCCAGGGGGTTTTTTAAAATTGGGGGGGGTAAAACAAAAACCCCCCCCCCCGGGGGGGTCCCCCAACCCCACCCCCCCCCCCCCGGGGGGGGGTGCGGGGGGAAATATTT

>JAG2

TGCCGTTTGTGGTGGGTTCTAATATGTATGTGTGTATCTAAACTTATATTGTCCACATGGACATGGACATGGACTGGTACCCAATGGTATTGATGCTATGTGTATATCAACCTCCGCATATTTTCTCCCTGTTGACCACGGCTCCCGGAACGTGTCGCGATGGATGACTTGGCTTCCTATTTCGTTGAAGAACGCAGCAAAGTGC

>JAD7

GTCATCACAAGGTATAAATCATCAAACCAGGAATGAAGGAGGGTAGTTCGGGGAGACGTACTTCTTAAGGGGTTTGTGCGGTTTAACGGGAATATCCTCAGCACGTTGTTTACATTTTTAGGTTACAGTCTCAGGGGGGAGTACGTTCGCAAGAGTGAAACTTAAAGAAATTGACGGAATGGCACCACAAGACGTGGAGCGTGCGGTTTAATTTGACTCAACACGGGGAACTTTACCAGATCCGGACAGGGTGAGGATTGACAGATTGAGTGTTCTTTCTCGATCCCCTGAATGGTGGTGCATGGCCGCTTTTGGTCGGTGGAGTGATTTGTTTGGTTGATTCCGTCAACGGACGAGATCCAAGCTGCCCAGTAGGGCCCGTGATTGTCCACACAGGACAGCCTACCGTCGTGGGCACGGTGTGTCACGCGAAAGCTTTGAGGTTACAGTCTCAGGGGGGAGTACGTTCGCAAGAGTGAAACTTAAAGAAATTGACGGAATGGCACCACAGGTGCGTCAGAGGTGAAATTCTTAGACCGCACCAAGACGAACTACAGCGAAGGCATTCTTCAAGAT
