# Supplementary material for: Molecular identification and prevalence of trypanosomes in cattle distributed within the Jebba axis of the River Niger, Kwara state, Nigeria
Source: Parasit Vectors. 2021 Oct 29;14:560. doi: 10.1186/s13071-021-05054-0 (PMC8557008; doi:10.1186/s13071-021-05054-0)
Supplement: Supplementary file 5 — Additional file 5: Table S4. Prevalence of Trypanosoma species infection among cattle in Jebba, Kwara State, Nigeria (June 2019). [file 13071_2021_5054_MOESM5_ESM.docx]

**Table S4: Prevalence of *Trypanosoma* species infection among cattle in Jebba, Kwara State, Nigeria (June 2019).**

| **Category** | **Type of Infection** | **PCV (Mean±SE)** | **No of Animal Infected** | **Specie Prevalence (%)** | **Overall Prevalence (%)** |
| --- | --- | --- | --- | --- | --- |
|  |  |  |  |  |  |
| Single infection |  | \|  \| \| --- \| |  |  |  |
|  | *T. congolense* | \| 23.8±1.15 \| \| --- \| | 6 | 50.00 | 91.67 |
|  | *T. brucei* | \| 30.3±0.92 \| \| --- \| | 2 | 16.67 |  |
|  | *T. evansi* | 20.2±0.32 | 1 | 8.33 |  |
|  | *T. theileri* | 19.2±1.12 | 1 | 8.33 |  |
|  | *T. simiae* | 22.0±0.63 | 1 | 8.33 |  |
|  |  |  |  |  |  |
| Mixed infection |  |  |  |  |  |
|  | *T. congolense + T. vivax* | 24.2±0.23 | 1 | 8.33 | 8.33 |
|  |  |  |  |  |  |
| Total |  | 23.2±2.63 | 12 | 100.0 | 100.0 |
