# Supplementary material for: Molecular identification and prevalence of trypanosomes in cattle distributed within the Jebba axis of the River Niger, Kwara state, Nigeria
Source: Parasit Vectors. 2021 Oct 29;14:560. doi: 10.1186/s13071-021-05054-0 (PMC8557008; doi:10.1186/s13071-021-05054-0)
Supplement: Supplementary file 6 — Additional file 6: Table S5. Prevalence of Trypanosoma congolense infection (according to types: Kilifi, Savannah, and Forest), among cattle in Jebba, Kwara State, Nigeria (June 2019) [file 13071_2021_5054_MOESM6_ESM.docx]

**Table S5: Prevalence of *Trypanosoma congolense* infection (according to types: Kilifi, Savannah, and Forest), among cattle in Jebba, Kwara State, Nigeria (June 2019)**

| **Type of infection** | **PCV (MEAN±SE)** | **No of animal infected** | **Prevalence (%)** |
| --- | --- | --- | --- |
|  |  |  |  |
| *T. c.* Kilifi | 23.2±0.58 | 1 | 14.3 |
| *T. c.* Savannah | 22.6±0.26 | 5 | 71.4 |
| *T. c.* Forest | 24.4±0.37 | 1 | 14.3 |
| Total | 23.6±1.85 | 7 | 100.0 |
